# Structure-driven function of plant lncRNAs: conserved RNA architectures in transcriptional and post-transcriptional regulation

**DOI:** 10.1080/15476286.2026.2664959

**Published:** 2026-04-27

**Authors:** Yanqi Shen, Qianli Dong, Yiliang Ding, Huakun Zhang

**Affiliations:** a Key Laboratory of Molecular Epigenetics of the Ministry of Education, Northeast Normal University, Changchun, China; b Department of Cell and Developmental Biology, John Innes Centre, Norwich Research Park, Norwich, UK

**Keywords:** Long noncoding RNA (lncRNAs), RNA secondary structure, plant, transcription, translation, RNA stability

## Abstract

Long noncoding RNAs (lncRNAs) have emerged as critical regulators of plant development, physiology, and environmental stress adaptation. Despite exhibiting limited primary sequence conservation across the plant kingdom, emerging evidence underscores that plant lncRNA functionality is encoded at the level of RNA secondary structure, facilitating diverse modes of structure-mediated gene regulation. In this review, we synthesize recent advances in elucidating the structural conservation, and molecular functions of plant lncRNAs. We further discuss their expanding regulatory repertoire and assess their potential utility as innovative molecular tools for crop improvement.

## Introduction

Long noncoding RNAs (lncRNAs) are transcripts longer than 200 nucleotides that lack significant protein-coding potential [1,2] and are widely present across eukaryotic genomes [1,2]. In plants, lncRNAs participate in numerous critical biological processes, including regulation of flowering-time [3–6], sexual reproduction [7], seed development [8], responses to abiotic and biotic stresses [9–12], and epigenetic silencing [5,13,14]. Based on their genomic context relative to protein-coding genes, plant lncRNAs are categorized into subtypes such as long intergenic noncoding RNAs (lincRNAs), natural antisense transcripts (NAT-lncRNAs), and intronic lncRNAs (incRNAs) [2,15]. Most plant lncRNAs are transcribed by RNA polymerase II and undergo splicing [16], although they generally contain fewer exons than mRNAs, with those exons typically being longer in length [17]. Some lncRNAs also exhibit tissue-specific or stress-responsive expression, suggesting tightly regulated transcriptional control and functional specialization.

Unlike protein-coding genes, plant lncRNAs show minimal primary sequence conservation, even among closely related species [18], posing significant challenges for sequence-based functional annotation. This limitation has shifted research from a ‘sequence-centric’ to a ‘structure-centric’ paradigm, recognizing that lncRNA function is largely encoded in secondary and tertiary structures [5,19–21]. RNA structure not only influences transcript stability, but it also dictates specific interactions with DNA, proteins, and other RNAs, forming the structural basis for lncRNA-mediated regulation. More broadly, accumulating evidence indicates that lncRNA activity may be largely mediated through structurally driven interactions within the lncRNA interactome. For instance, the lncRNA *EARLY NODULIN40* (*ENOD40*) interacts with the RNA-binding protein 1 (RBP1) in *Medicago truncatula* [22], while in *Arabidopsis thaliana*, NSR proteins (homologs of RBP1) associate with the lncRNA *ALTERNATIVE SPLICING COMPETITOR* (*ASCO*) [23,24]. *ASCO* also interacts with core spliceosomal components such as *PRP8* and *SmD1b*, and NSR proteins bind multiple lncRNAs genome-wide [25]. Notably, despite the lack of sequence similarity between *ENOD40* and *ASCO*, *ENOD40* can interact with MtNSR and relocalize it to the cytoplasm, highlighting a structure-dependent mode of interaction [22]. Similarly, the flowering-associated intergenic lncRNA (*FLAIL*) has been reported to associate with splicing factors [26]. Together, these findings support a model in which lncRNAs without conserved sequences may converge on shared protein partners through common structural features, reinforcing the central role of RNA structure in shaping the lncRNA interactome.

Recent advances in *in vivo* RNA structure probing technologies - including dimethyl sulphate (DMS) profiling (Structure-seq, DMS-MaPseq), SHAPE chemical probing approaches (SHAPE-Structure-seq, icSHAPE, SHAPE-MaP), and single-molecule structure sequencing (smStructure-seq) - have enabled high-resolution mapping of RNA conformations under physiological conditions, revealing previously hidden structural heterogeneity and dynamic folding landscapes [19–21,27–33].

Although this review focuses on plant systems, particularly the dicot *Arabidopsis* and the monocot crop wheat (*Triticum aestivum*), the fundamental principle that RNA structure underlies function extends across eukaryotes. In mammals, lncRNAs such as *Xist* (a ∼17–19 kb lncRNA from the future inactive X, drives X-chromosome inactivation) and *HOTAIR* (HOX transcript antisense intergenic RNA) similarly rely on conserved structural domains to exert regulatory roles [34,35], emphasizing the evolutionary conservation of RNA architecture in diverse regulatory networks. In the following sections, we synthesize current knowledge on structural conservation in plant lncRNAs and examine how RNA structural features influence transcriptional, translational, and post-transcriptional regulation, drawing on animal examples to highlight shared mechanistic themes and potential cross-kingdom parallels.

## Structural conservation of plant lncRNAs

Although plant long noncoding RNAs (lncRNAs) typically evolve rapidly at the level of primary sequence, growing evidence demonstrates that their secondary and higher-order structures are subject to strong evolutionary constraint. One compelling example in plants is *COOLAIR*, a set of alternatively processed antisense long noncoding RNA transcripts generated from the *Arabidopsis* floral repressor locus FLOWERING LOCUS C (FLC), where they play key roles in the regulation of flowering time [36]. Despite the low overall sequence identity, multiple structural elements - including conserved stem-loops such as H3 (Helix 3) and H7, as well as complex multi-way junctions - are robustly maintained in the *in vitro* RNA structure profiles [5]. Strikingly, these conserved structures often coincide with splice sites or predicted protein-binding regions, suggesting that RNA architecture directly coordinates RNA processing and regulatory interactions [5]. These findings support a ‘sequence drift, structure retention’ model, in which selective pressure acts primarily to preserve functional RNA conformations rather than specific nucleotide sequences.

The functional relevance of such structural conservation is illustrated by the H8–H9 region of *COOLAIR*, which overlaps with a coding exon of FLOWERING LOCUS C (FLC). This region exhibits not only moderate sequence conservation but also retains of a distinctive right-handed turn motif across species [5]. Importantly, these insights would be difficult to derive from sequence analysis alone and are often obscured by conventional *in silico* RNA folding approaches, which typically predict a single minimum free-energy structure under simplified and static conditions.

**Figure 1.**
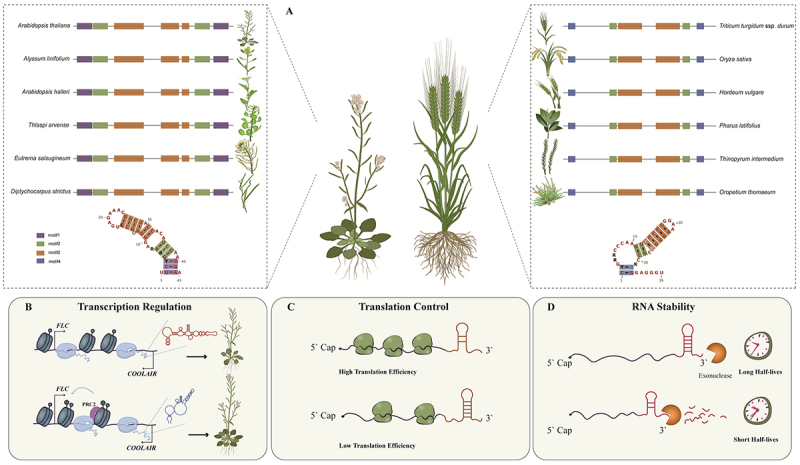
Conserved structures and functions of mechanisms of plant long noncoding RNAs in multilayered gene regulation. (A), Conserved RNA secondary structure of plant lncRnas across species. For both the monocot *Arabidopsis thaliana* and the dicot *Triticum aestivum*, lncRNA sequences exhibited structural conservations across individual evolutionary clades. (B), lncRNA structure functions in regulating transcription. The schematic illustrates the changes of *Arabidopsis* antisense lncRNA COOLAIR structures that promote flowering. (C), lncRNA structure functions in affecting translation. The translation-associated structural motifs are located at their 3′ ends. (D), lncRNA structure functions in influencing RNA stability. The stability of lncRnas is governed by structural motifs located at their 3′ ends.

Comparative analyses across Solanaceae further support a structure-centric conservation model: Although primary sequence conservation is low, synteny, structural motifs, and functional correlation remain detectable, particularly among lincRNAs [37]. Genome-wide *in vivo* structurome profiling confirmed that 33% of *Arabidopsis* and 60% of wheat lncRNAs harbour at least one conserved RNA structural motif, with these motifs preferentially adopting stable folds [38] (Figure 1(A)). Notably, wheat lncRNAs are longer, GC-richer, and more structurally stable than those in *Arabidopsis*, providing robust cross-species evidence for structure-driven conservation [38].

The phenomenon is also well documented in animals. In mammals, the PRC2-binding module of *HOTAIR* maintains a conserved hairpin despite sequence divergence [35]. *Xist* drives X-chromosome inactivation by coating the chromosome, which contains Repeat A, using a conserved stem-loop [39]. Collectively, these findings indicate that structural conservation is a fundamental evolutionary strategy for encoding lncRNA function. However, the prevalence of whole-genome duplication in plants complicates functional dissection, emphasizing the need for integrated genetic and multi-omics approaches.

## Structural roles of lncRNAs in transcriptional regulation

A major conceptual advance arose from single-molecule *in vivo* smStructure-seq, which resolved structural heterogeneity across individual *COOLAIR* isoforms [20]. In particular, it provided critical insight into the structural organization of class II.i *COOLAIR*. Rather than adopting a single dominant fold, *COOLAIR* was shown to populate at least three distinct and well-defined structural conformations within living cells [20]. These conformations are characterized by alternative arrangements of conserved helices and junctions, including differential pairing within central stem–loop regions and reorganization of multi-way junctions that likely alter the accessibility of protein-binding surfaces. Importantly, these conformations coexist simultaneously under normal growth conditions, demonstrating that structural heterogeneity is an intrinsic and stable property of *COOLAIR* rather than a transient folding intermediate or stochastic noise [20].

The *COOLAIR* structure is further shown to be dynamically responsive to environmental signals. Upon cold exposure during vernalization, smStructure-seq detected the emergence of an additional, cold-specific structural conformation, accompanied by shifts in the relative abundance of the pre-existing conformational states [20]. This remodelling reflects coordinated structural changes across the entire structural landscape, rather than local unfolding, indicating a global reorganization of RNA architecture. Such environmentally induced redistribution of structural states suggests that temperature cues reshape the *COOLAIR* conformational landscape.

The functional importance of these defined RNA structures is underscored by genetic analyses. Mutations that enlarged the bulge within the H4–H6 region by shortening helices H4 and H5 increased chromatin association of class II *COOLAIR* across the FLC transcription start site (TSS) region in mutant plants. This enhanced chromatin association was accompanied by reduced transcriptional output from the FLC locus and a corresponding acceleration of flowering time [20]. These results demonstrate that *COOLAIR* activity is encoded at the level of RNA structure rather than primary sequence, and that precise folding of defined structural domains is required for proper transcriptional repression (Figure 1(B)). The *COOLAIR* conformations might be differentially engaged with RNA-binding proteins, splicing factors, or chromatin-associated regulators, thereby influencing co-transcriptional processing and chromatin state at the FLC locus.

Similar lncRNA-mediated mechanisms of transcriptional regulation have been described in diverse developmental and environmental contexts. Under far-red light, the lncRNA *AUXIN-REGULATED PROMOTER LOOP* (*APOLO*) modulates chromatin architecture to repress *BRC1* and increase shoot branching [9]. *DANA2* positively regulates drought response by co-regulating *JMJ29* transcription with ERF84 [40], while hyperoside-induced lncRNAs *lnc187* and *lnc999* promote pollen tube growth and seed set in pigeonpea [41]. In wheat, the antisense lncRNA *VAS* interacts with the transcription factor TaRF2b to activate *TaVRN1* and control vernalization-responsive flowering [42]. These examples suggest that lncRNA-mediated transcriptional regulation is widespread in plants and that, as in *COOLAIR*, structured RNA domains may contribute to their regulatory functions, although the extent of mechanistic conservation remains unclear. Whether these lncRNAs operate through shared or distinct molecular mechanisms remains to be determined. In addition to chromatin regulators and RNA-binding proteins, transcription factors (TFs) can directly interact with lncRNAs, adding another layer of regulation. For example, *APOLO* interacts with WRKY42 to modulate gene expression [9,43]. More broadly, many TFs exhibit RNA-binding capacity [44], raising important mechanistic questions regarding how TFs distinguish between DNA and RNA substrates. These observations suggest a potential structural similarity between DNA and RNA substrates in their recognition by TFs.

These regulatory mechanisms have clear parallels in animal systems. An interesting cross-kingdom example is provided by the human lncRNA *UPAT*. When expressed in *Arabidopsis*, *UPAT* interacts with *APOLO*-associated proteins such as VIM1 and LHP1 and phenocopies *APOLO* overexpression [45,46]. Notably, *UPAT* also binds the mammalian homolog UHRF1 despite lacking sequence similarity with *APOLO* [45]. This supports the idea that conserved structural features, rather than primary sequence, may mediate lncRNA – protein interactions across evolutionary distances. *Xist* uses its repeat structural domains to recruit chromatin modifiers, initiating and maintaining transcriptional silencing [34]. *HOTAIR* functions as a modular scaffold to regulate gene transcription by recruiting chromatin-modifying complexes to specific loci, with its structure-dependent activity relying on independently folded 5’and 3’ domains to coordinate epigenetic silencing [35]. Furthermore, *MALAT1* (Metastasis Associated Lung Adenocarcinoma Transcript 1) is an ~ 8 kb lncRNA that regulates transcription and splicing in a structure-dependent manner by acting as a nuclear scaffold that organizes genome architecture and recruits regulatory proteins to chromatin [47]. *Neat1* (Nuclear Enriched Abundant Transcript 1) is a long non-coding RNA (lncRNA) that regulates gene expression in a structure-dependent manner. Its long isoform, NEAT1_2 (~23 kb), is essential for paraspeckle assembly, adopting a core-shell architecture in which the central region scaffolds paraspeckle-associated proteins (PSPs) to nucleate and stabilize these subnuclear bodies, whereas the short isoform, NEAT1_1 (~3.7 kb), is dispensable [48]. Across kingdoms, lncRNAs thus employ RNA structural strategies to interface with chromatin-modifying complexes—a testament to the deep evolutionary logic of RNA-guided epigenetic regulation.

## Structural determinants of translational regulation

Beyond transcriptional control, RNA structure also governs the translational potential of plant lncRNAs. Although most lncRNAs are noncoding, they can directly or indirectly influence translational efficiency, with RNA secondary structure serving as a central determinant.

A systematic study by Dong et al., employing multi-omics analyses in *Arabidopsis* and wheat, provided the first comprehensive landscape of plant lncRNA regulation at the post-transcriptional level [38]. This study demonstrated that RNA secondary structure may serve as a central factor controlling the translational regulation of lncRNAs: lncRNAs with more open/less structured regions (indicated by higher SHAPE reactivity and lower base-pairing probability) generally exhibited higher translation efficiency, suggesting that single-stranded, accessible conformations facilitate ribosome loading [38]. Moreover, in these two evolutionarily distant species, RNA structural motifs significantly associated with translation were systematically identified, and these motifs were notably enriched at the 3’ ends of lncRNA transcripts [38] (Figure 1(C)). This conserved spatial localization pattern across species implies that 3’ terminal structures may play a key role in recruiting the translation machinery or regulating ribosome readthrough. While some lncRNAs in *Arabidopsis* have been reported to produce functional micropeptides involved in stress responses, the functional significance of such phenomena in crops like wheat and cotton remains largely exploratory [9,49–51].

In animals, lncRNAs also modulate translation through diverse structural mechanisms. In human HeLa cells, reduced HuR levels lead to the accumulation of *lincRNA-p21*, which mediates translational inhibition of downstream target mRNAs—for instance, *CTNNB1* [52]. In stressed mouse hearts, the ribosome-associated lncRNA *CARDINAL* binds DRG1 to disrupt its interaction with DFRP1, thereby tuning global translation rates [53]. These examples underscore the versatility of lncRNA-mediated translational control across eukaryotes.

## Structure functions in influencing RNA stability

RNA stability is a crucial post-transcriptional checkpoint determining gene expression levels, and RNA secondary structure plays a central role in this process. Recent research indicates that this regulatory mechanism applies not only to mRNAs but also broadly to lncRNAs [54–56]. For instance, plant lncRNAs tend to form complex secondary structures, potentially minimizing exposure of single-stranded regions and thereby reducing the risk of recognition and degradation by nucleases [55]. Structural stability (e.g. regions rich in double-stranded elements) generally correlates positively with longer half-lives, while lncRNAs with more open structures are more rapidly turned over [38]. Notably, although the primary sequences of lncRNAs are often poorly conserved across species, their secondary structures can show significant conservation within specific lineages (e.g. Brassicaceae or Poaceae), suggesting that the structure itself may carry critical functional information [38,56].

Unlike mRNAs, plant lncRNA stability is particularly governed by RNA structural motifs enriched in the 3’ end of lncRNAs. Specific RNA structural motifs (e.g. stable RNA Structural Motifs, sRSMs) can prolong lncRNA half-lives by impeding exonucleases or recruiting protective RNA-binding proteins, whereas unstable RNA Structural Motifs (uRSMs) may accelerate their degradation [55] (Figure 1(D)). For example, wheat lncRNAs, characterized by higher GC content and more stable folding conformations, exhibit a significantly longer average half-life (~10.5 hours) compared to *Arabidopsis* lncRNAs (~2. 3 hours), underscoring the profound impact of structure on stability [55].

Some structural motifs within lncRNAs are highly conserved throughout evolution, indicating their critical importance for function [57]. These conserved structures may maintain lncRNA stability and ensure proper function by stabilizing the RNA conformation [58,59], protecting key functional regions, or facilitating interactions with other molecules [60,61].

Conserved structural elements also play essential roles across eukaryotes. The mammalian lncRNA *NORAD* employs conserved stem-loop structures to bind PUMILIO proteins with high affinity, stabilizing itself while preventing excessive suppression of mRNAs related to genomic stability [62]. An enhancer-derived lncRNA near *NPAS4* promotes chromatin looping through R-loop formation, accelerating *NPAS4* expression and indirectly influencing RNA stability [63]. Together, these findings collectively demonstrate that RNA secondary structure may serve as a determinant of lncRNA stability.

## Conclusions and perspectives

A major strength of this work is its ability to connect mechanisms observed in plants with those characterized in other eukaryotes, highlighting both conserved principles and lineage-specific adaptations. Across kingdoms, RNA structure underpins lncRNA function, yet differences in plant biology can lead to distinct regulatory outcomes. For example, plants frequently undergo whole-genome duplications and extensive polyploidy, which may increase the reliance on lncRNAs for fine-tuning gene expression and allow structurally conserved lncRNAs to acquire novel targets or functions. In addition, plants face unique environmental cues—such as vernalization, photoperiod sensing, and abiotic stress—that can drive dynamic structural rearrangements in lncRNAs, as exemplified by *COOLAIR* and *APOLO*, whereas analogous animal lncRNAs more often modulate development or dosage compensation. These differences suggest that plant lncRNAs may have evolved highly adaptable structural modules to integrate external signals with transcriptional and post-transcriptional regulation, a feature that may be less pronounced in more developmentally constrained animal systems.

At the same time, many structural strategies are shared across kingdoms: modular scaffolding, recruitment of chromatin modifiers, and the formation of stable secondary and tertiary motifs, as seen in *Xist*, *HOTAIR*, and *MALAT1*, reflect a deep evolutionary logic. These parallels suggest that additional plant lncRNAs may employ yet-undiscovered structural domains or recruitment mechanisms analogous to those characterized in animals, such as m^6^A-mediated regulation, higher-order nuclear body formation, or phase-separated compartments. Moreover, the interplay between RNA structure and protein cofactors, as well as RNA–RNA interactions, may provide an additional layer of regulation that remains largely unexplored in plants.

The functional roles of plant lncRNAs are largely dictated by their dynamic and multilayered structures. Studies of model lncRNAs like *COOLAIR* demonstrate that RNA structure serves not merely as molecular ‘armor’ for stability but also as a functional ‘interface’ for sensing environmental signals and mediating gene regulation. Despite the low primary sequence conservation, critical structural domains are evolutionarily preserved, enabling precise regulation of transcription, translation, and RNA stability through conformational changes. This ‘structure-centric’ paradigm also holds true in animal systems, underscoring a deep unity in eukaryotic RNA regulatory mechanisms. Collectively, these insights emphasize that structure, rather than sequence, often encodes functional information, a principle that could guide predictive modelling and rational design of lncRNA-based regulators.

Future research will require integration of chromatin biology, RNA structural analysis, RNA-focused large language models, and crop engineering to fully unravel and exploit lncRNA function. Key directions include:

1. Elucidating how lncRNA structural elements shape chromatin architecture to regulate transcription.
2. Leveraging RNA large language models [19,64] to systematically identify conserved and functional structural motifs across diverse lncRNAs.
3. Harnessing RNA structure–guided engineering strategies to improve crop traits.

As high-resolution RNA structure landscapes emerge across major crop species, the prospect of engineering lncRNA structural modules to enhance agronomic traits—such as stress tolerance, flowering time, and yield—becomes increasingly attainable. In the longer term, integrating these mechanistic insights with synthetic biology approaches may enable the design of bespoke lncRNAs capable of modulating complex traits or buffering environmental fluctuations. Together, these efforts will deepen our mechanistic understanding of RNA-mediated gene regulation and provide a conceptual and technological foundation for the next generation of precision molecular breeding.

## Data Availability

No data were generated in this work.
